# Improving Access to Home Blood Pressure Monitors at a Federally Qualified Health Center

**DOI:** 10.1177/87551225231156741

**Published:** 2023-03-11

**Authors:** Isha Deshpande, Amrita Kanwar, Kendra Swyers, Aida Garza, Kathryn Litten

**Affiliations:** 1 The University of Texas at Austin College of Pharmacy, Austin, TX, USA; 2 CommUnityCare Health Centers, Austin, TX, USA

**Keywords:** home blood pressure monitor, self-monitoring of blood pressure, increasing access to care, hypertension, health equity

## Abstract

**Background:** Self-monitoring of blood pressure (BP) clinically decreases BP. However, cost can limit access, especially in underserved populations. **Objective:** This mixed-methods pilot study aims to determine the impact of providing home BP monitors free of charge to patients at a federally qualified health center (FQHC) by quantifying the effect on BP and surveying patients to measure satisfaction and engagement. **Methods:** One hundred eighty patients with clinically diagnosed hypertension received BP monitors. Patient charts were reviewed to collect demographics and office BP readings 3 months before and after receiving a monitor. A 13-question phone survey was conducted to a sample of patients addressing satisfaction and engagement. Answers were based on a Likert scale and dichotomous yes/no. Results were analyzed with descriptive statistics and paired *t* tests. **Results:** The chart review demonstrated a significant mean decrease in systolic BP by 5.44 mm Hg (*P* < 0.001, −8.03 to −2.84) and a mean decrease in diastolic BP by 2.70 mm Hg (*P* < 0.001, −4.08 to −1.32) after the intervention. For those included who responded to the survey (13%), there was a significant mean increase in the frequency of checking BP per week by 1.5 Likert points (*P* < 0.00001, −1.0 to −1.9), and a majority (57.8%) felt slightly or much more active in their health care in addition to other benefits. **Conclusion:** Providing BP monitors to FQHC patients free of charge may have contributed to a significantly decreased office BP, improved engagement, and satisfaction. This program removed cost barriers and allowed patients to be more active in their health care.

## Background

Hypertension (HTN) is defined by the American Heart Association as a systolic blood pressure (SBP) greater than 130 mm Hg or a diastolic blood pressure (DBP) greater than 80 mm Hg.^1^ Nearly 45% of adults in the United States are diagnosed with HTN, but only 1 in 4 have their condition controlled.^2^ Hypertension is a leading risk factor for ischemic and hemorrhagic strokes, heart attacks, heart failure, aneurysms, kidney damage, changes in vision, and dementia.^1^ The American College of Cardiology/American Heart Association (ACC/AHA) guidelines recommend a goal of lowering the blood pressure (BP) to ≤130/80 mm Hg for most patients to lower the risk of serious complications.^1^ Methods to lower BP include lifestyle changes such as salt reduction, exercise, and antihypertensive medications. Increasing patient’s awareness of their condition and BP through education and engagement may also decrease BP.^3,4^

Office readings only provide a brief glimpse at a patient’s overall HTN control. Self-monitoring blood pressure (SMBP), or home monitoring, is a tool that helps patients with HTN monitor BP outside of the office, providing more data for their provider to make clinical decisions. Many BP guidelines recommend out-of-office monitoring to confirm a diagnosis of HTN or differentiate white coat and masked HTN. ACC/AHA guidelines recommend SMBP to help make pharmacotherapy interventions.^1,5^ In 2008, the AHA issued a call to action on the use of and reimbursement for home BP monitoring, highlighting its benefits, including allowing patients to track treatment outcomes independently in order to motivate them to have better control.^6^ Trials have shown that SMBP, in conjunction with usual therapy, led to clinically meaningful improvements in both SBP and DBP. A Cochrane review found that SMBP reduced SBP by 2.5 mm Hg and DBP by 1.8 mm Hg.^7,8^ This practice may lead to decreased office visits, complications, and hospitalizations.^9^ These benefits support a need to increase SMBP.

Patients who are socially or economically disadvantaged have a higher risk of HTN. This population may particularly benefit from SMBP to overcome medical access barriers such as lack of transportation to clinic appointments. However, there still remain obstacles to SMBP implementation, such as out-of-pocket costs of the BP monitor and compliance.^5,10-13^ In order to overcome these barriers and improve access to health services per the Healthy People 2030 goal, CommUnityCare Health Centers, a federally qualified health center (FQHC) in Austin, Texas, implemented a program to distribute BP monitors free of charge to patients without insurance who had a clinical diagnosis of HTN. Patients were able to pick up the BP monitor from the pharmacy or have it delivered to their home to limit transportation barriers. This program was conducted in conjunction with the Travis County HealthCare District, a taxing entity with a mandate to provide health care to Travis County’s indigent or uninsured population. To determine the sustainability of the program, our research served to quantify changes in BP before and after the patients received a BP monitor and to survey patients for satisfaction and engagement with the home BP monitor. We hypothesized that by overcoming health access barriers and providing BP monitors free of charge, we are able to increase data sources for providers to improve clinical inertia and increase patient engagement into care, both contributing to better patient outcomes.

## Objectives

This mixed-methods study at a FQHC aimed to determine the impact of providing home BP monitors to patients free of charge by quantifying the effect on in-office BP readings after the patients received a BP monitor and surveying patients to measure satisfaction and engagement qualities.

## Methods

Pharmacy claims data were utilized to identify patients with clinically diagnosed HTN who received BP monitors from May through September 2020. A chart review was conducted to collect demographics (race, gender, and age) and up to 3 most recent in-office BP readings both 3 months before and after receiving a monitor. Patients who had at least 1 BP reading before and after receiving the monitor were included in the study. The pre-SBP and pre-DBP readings were averaged for each patient, and the same was done for the postreadings. The clinical metric targets for BP was defined as <140/<90 mm Hg, as defined by the Delivery System Reform Incentive Payment (DSRIP) of Medicaid Waiver 1115, and <130/<80 mm Hg as the BP goal for most patients per the ACC/AHA guidelines. A paired *t* test was performed on the average office BP readings, and confidence intervals with an alpha value of 0.05 determined the significance of the results. This study was exempt by the institutional review board at the University of Texas at Austin.

A 13-question survey was developed addressing patients’ satisfaction and engagement with the BP monitor, adherence to their HTN medications, and motivation in their health care since having the BP monitor (Appendix A). Answers were based on a Likert scale or a dichotomous yes/no. Surveys were distributed via phone by pharmacy students and a bilingual technician (English and Spanish) from May 2021 to June 2021. Participants were excluded if they responded they had not received a BP monitor, declined the survey, or did not answer after 2 phone call attempts. Survey responses were documented in Qualtrics (Seattle, Washington). Likert scales were analyzed using descriptive statistics. For survey questions that compared the number of times BP was taken before and after receiving the monitor, a 1-sided paired *t* test was used to analyze the significance with an alpha value of 0.05.

## Results

### Data Analysis

One hundred and eighty patients received a monitor during the trial period, and 145 met inclusion criteria for participation in the survey based on the chart review. Others were excluded due to a lack of recorded in-office BP reading. Of the 145 patients, 57% were female, and the average age was 60 years. The race distribution was 68% Hispanic, 12% black, 8% white, 5% Asian, and 9% did not report. Attempts were made to call all 145 patients. Three did not have a phone number listed, 23 patients declined, 6 stated they had not received a monitor, and 94 patients were not reachable due to no responses for phone calls after two attempts. Nineteen patients completed the survey. Characteristics of survey respondents were similar to those of the chart review patient group with the exception of race. Notably, none of the survey population identified as Hispanic. The baseline demographics are given in Table 1.

**Table 1.** Baseline Characteristics for Chart Review and Survey Patient Populations.

	Chart review	Survey
Characteristic	No. (%)	No. (%)
**Total N**	145	19
**Age**		
≤50	36 (25)	7 (37)
>50	109 (75)	12 (63)
Mean (SD)	60.4 (16.09)	53.0 (9.58)
**Sex**		
Male	62 (43)	8 (42)
Female	83 (57)	11 (58)
**Ethnicity^a^**		
Hispanic	98 (68)	0 (0)
Black	17 (12)	4 (21)
White	12 (8)	5 (26)
Asian	7 (5)	1 (5)
Unreported	13 (9)	9 (47)
**Primary language**		
English	65 (45)	17 (89)
Spanish	71 (49)	2 (11)
Other^b^	4 (3)	0 (0)
Unknown	5 (3)	0 (0)
**Blood pressure**		
≤140/90 mm Hg	82 (57)	—
>140/90 mm Hg	62 (43)	—
Overall mean (SD)	140/80 (16.4/10.0)	

a Some patients reported 2 ethnicities and were counted in both groups. The percentages total up to more than 100% because of this.

b Other languages include Korean, Farsi, and Arabic.

#### Impact on BP

The results of BP changes are shown in Table 2. There was a significant mean decrease in SBP by 5.44 mm Hg (*P* < 0.001, −8.03 to −2.84) and a mean decrease in DBP by 2.70 mm Hg (*P* < 0.001, −4.08 to −1.32). Before the intervention, only 57% of patients were at the clinical target of ≤140/90 mm Hg. After the intervention, 73% of patients were at the clinical target, an absolute increase in number of patients at goal of 16%. More patients met the ACC/AHA guideline target, with a majority of patients with BP <130/80 mm Hg meeting the target by the end of the study as well, with only 20% (29) of <130/80 mm Hg before the intervention and 30% (44) after the intervention (change in SBP *P* < 0.001, DBP *P* = 0.002).

**Table 2.** Average Change in Office Blood Pressure and Home Monitoring Frequency.

	Before (SD)	After (SD)	Difference	*P* value (CI)
Office BP readings				
Average systolic BP (mm Hg)	140 (16.4)	134 (16.2)	−5.44	<0.001 (−8.03 to −2.84)
Average diastolic BP (mm Hg)	80 (10.0)	78 (10.0)	−2.70	<0.001 (−4.08 to −1.32)
Patient survey				
Change in frequency of home BP monitoring (Likert point)	1.5 (0.90)	3.0 (1.15)	−1.5	<0.00001 (−1.0 to −1.9)

Abbreviations: BP, blood pressure; CI, confidence interval.

#### Impact on engagement and satisfaction

The full survey and responses can be found in Appendix A. The response rate was 13% (19/145). The survey results for the first 2 questions, addressing how often patients checked their BP, are shown in Table 2. Before receiving their BP monitors, patients reported checking their BP less than once a week on average. After receiving their BP monitors, this average increased to checking their BP multiple times a week. This is represented by an increase in 1.5 points on the Likert scale (1, less than once a week; 2, once a week; 3, a couple of times a week; 4, once a day; 5, multiple times a day). Table 3 highlights other engagement and satisfaction survey results (see Abstract for full survey results).

**Table 3.** Select Engagement and Satisfaction Survey Results Following Receipt of Blood Pressure Cuff.

Responses (percent), n = 19	Response
15 (78.9%)	They did not need any more instruction on using the BP monitor
17 (89%)	BP readings had been at goal at home
17 (89%)	Feeling somewhat or extremely motivated to take steps to lower their BP throughout the day, such as eating healthier, limiting salt, exercising, etc.
4 (21%)	Remembering to take their BP medications better There was largely no change in remembering to take other chronic medications
11 (57.8%)	Feeling slightly or much more active in their health care
16 (84.2%)	Feeling satisfied or very satisfied with their BP monitor
14 (73.7%)	They were likely or very likely to follow up with their provider now that they have an at-home BP monitor.
12 (63%)	They are extremely likely to bring their BP readings to their provider

Abbreviation: BP, blood pressure.

## Discussion

Increasing access to a home BP monitor resulted in a significant decrease in both SBP and DBP. Seventy-three percent of patients met the clinical metric, an absolute improvement of 16%. In addition to clinical metrics, providing BP monitors significantly increased the frequency at which patients checked their BP, improving patient engagement with their health. Patients reported feeling more motivated, more likely to follow up with their provider, and they were satisfied with the program. This study focused on both aspects to better determine clinical significance. Previous studies involving home monitoring devices have either focused on quantitative BP changes or assessing patient satisfaction, but not both.^10-15^ The study’s mixed-methods structure was a strength to provide the FQHC with valuable feedback from patients on the benefits of expanding this program in addition to a significant primary endpoint.

A few main themes likely led to the significant improvement in BP. This project succeeded in overcoming the cost barrier for patients obtaining a BP monitor, allowing those with little money to spend on ancillary products the chance to engage in their health. Transportation is a major obstacle for many patients, but by offering to deliver the monitor to the patient’s home, this barrier was eliminated as well. The impact of this intervention and the barriers it can overcome in the medically underserved population may have been 1 reason this study found a higher impact on SBP and DBP than previous trials assessing the impact of SMBP compared to a more general population (compared to the Cochrane review: SBP −5.44 vs −2.5 mm Hg and DBP −2.7 vs 1.8 mm Hg).^8^

This project happened to occur when the COVID-19 pandemic was at its height and primary care transitioned to largely telehealth appointments. Hypertension is often asymptomatic, so without a BP reading, there would be little to no information to assess control. If given a BP monitor, patients may have given providers more objective data to monitor, allowing them to make more informed decisions and medication adjustments to BP regimens without the patient coming to the clinic. Although provision of home readings and medication interventions were outside the scope of this study due to time and resources, this project may contribute to improving clinical inertia for patients unable to come to the clinic due to a number of reasons from transportation to international pandemic.

This study added to the body of research highlighting positive patient satisfaction and engagement in care with at-home BP monitoring.^10-13,15^ This survey data support that patients are more engaged by checking their BP more often and are more empowered to take action for their health. Although this survey showed a lower rate of patients reporting improved adherence to medications, it was still positive, and patients did feel more motivated to take additional steps such as therapeutic lifestyle changes. Further research in this population is needed to determine if it improved appointment follow-up rates.

One concern with providing these resources free of charge is sustainability. Although it has been shown that medical costs to the patient are decreased with the initiation of SMBP in physician visits, telephone calls, and laboratory tests,^11,12^ the health system must have the funds available to provide the monitor to patients. Federally qualified health centers and other systems providing care to underserved patients receive funding as part of the DSRIP of Medicaid Waiver 1115 for meeting pay-for-performance quality metrics. One metric is the number of patients with diabetes that have BP readings under 140/90 mm Hg.^16,17^ After the intervention, 16% more patients met this goal. Comorbidities were not collected as demographic data points for this study, so the number of patients who have diabetes and meet the DSRIP measure cannot be quantified. However, these data will likely lead to an impact given the high rate of comorbidity with HTN and diabetes. When applied to a larger population of patients, this may lead to an increase in funding for the site to continue to provide this service, and others, to directly benefit patients. Regardless, 10% more patients met the guideline-directed goal for most patients, <130/80 mm Hg, which can lead to reduction of complications and health care costs in long term.

Limitations of this study include the low number of overall patients included and lack of survey data in Spanish-speaking patients, who made up over half of the patients receiving the monitors. This was due to a lack of natural speakers and translator services available during the study time and contributed to sampling bias. Low numbers of patients reached by phone may be due to the transient nature of this population, another indication of the patient barriers to care. Patients may not have a phone, may share with others, or frequently change numbers, which significantly impacted the number of surveys gathered. These self-reported surveys were conducted up to a year after patients received the BP monitor, which could have also contributed to recall bias and low response rates. In addition, due to COVID-19, there were limited in-person appointments, and most follow-up BP readings were not recorded in a chart for telehealth appointments. Taking averages of multiple BP readings allowed for a more well-rounded clinical picture, but the data from these telehealth appointments could have been meaningful to the change in BP analysis and show a higher impact. During the survey, it was also noted that some patients did not receive the BP monitors documented as dispensed. Reasons may be due to others picking up the prescription for them or not understanding what the device is. Education to the patient about home BP monitoring was not documented in the chart and, therefore, likely varied among patients. Although most reported knowing how to use the monitor, having a specific educational plan for the patient may have further increased engagement and adherence. This is an opportunity for pharmacist or pharmacy technician involvement at the point of dispensing. Furthermore, at this clinic system, clinical pharmacists provide medication therapy management under a collaborative practice agreement in office or by telehealth appointments. Clinical pharmacy telehealth benefits for HTN are well documented in literature, so it would be interesting to determine if the intervention of providing BP monitors free of cost impacted the clinical pharmacy’s patient outcomes or increased interventions at this site.

This was a short-term pilot study that showed the positive impact in patient engagement, satisfaction, and BP for patients who received a BP monitor at no cost. For future considerations, patients should be evaluated over the course of a year to determine long-term effects of increased access to home BP monitors and if this ultimately decreases adverse events and/or hospitalizations.

## Conclusion

Providing BP monitors to FQHC patients free of charge may have contributed to the significant reduction to in-office BP, a significant increase in BP monitoring, and patient-reported overall improvement in patient engagement and satisfaction. Implementation of this program overcame the cost barrier of monitors and allowed patients to be more active in their care through SMBP. These benefits provide incentive for more health care facilities to adopt this service to support providing patient-centered, quality, and equitable care to all.

## Appendix A

**Table:** Survey Questions and Responses.

Question	Response	No. (%)
1. How often did you check your blood pressure before you got your new monitor?	Multiple times a day	0 (0)
	Once a day	1 (5.26)
	A couple of days a week	2 (10.53)
	Once a week	3 (15.79)
	Less than once a week	13 (68.42)
2. How often do you check your blood pressure now that you have a home monitor?	Multiple times a day	2 (10.53)
	Once a day	4 (21.05)
	A couple of days a week	7 (36.84)
	Once a week	4 (21.05)
	Less than once a week	2 (10.53)
3. Do you feel that you need more instruction on how to use your blood pressure monitor correctly?	Yes	5 (26.32)
	No	14 (73.68)
4. Have you noticed that your blood pressure readings have been at goal?	Yes	17 (89.47)
	No	2 (10.53)
5. What was your last blood pressure reading?	Average SBP	140.71 mm Hg
	Average DBP	83.15 mm Hg
	Did not remember	3 (15.7%)
6. Have you been more motivated to take steps to lower your blood pressure throughout the day? (eating healthier, less salt, exercising, etc.)	Very unmotivated	0 (0)
	Unmotivated	1 (5.26)
	Neutral	1 (5.26)
	Motivated	12 (63.16)
	Very motivated	5 (26.32)
7. In the past 2 weeks, how many days have you forgotten to take your blood pressure medication?	Never	11 (57.89)
	1-3	6 (31.58)
	4-7	0 (0)
	8-11	2 (10.53)
	12-14	0 (0)
8. Has forgetting to take your medication decreased since you have been using the blood pressure monitor?	Yes	4 (21.05)
	No	1 (5.26)
	No change	14 (73.68)
9. Does knowing your blood pressure make you more adherent to your other chronic medications?	Yes	5 (26.32)
	No	2 (10.53)
	No change	12 (63.16)
10. Do you feel satisfied with the blood pressure monitor?	Very dissatisfied	0 (0)
	Dissatisfied	1 (5.26)
	Neutral	2 (10.53)
	Satisfied	4 (21.05)
	Very satisfied	12 (63.16)
11. Do you feel more active in your health care/with your hypertension since you have received a blood pressure monitor?	Very unengaged	0 (0)
	Unengaged	0 (0)
	Neutral	8 (42.11)
	Engaged	5 (26.32)
	Very engaged	6 (31.58)
12. Are you any more likely to follow up with your provider now that you have an at-home blood pressure monitor?	Very unlikely	0 (0)
	Unlikely	0 (0)
	Neutral	5 (26.32)
	Likely	5 (26.32)
	Very likely	9 (47.37)
13. Are you going to bring your readings to your next appointment?	Very unlikely	1 (5.26)
	Unlikely	2 (10.53)
	Neutral	2 (10.53)
	Likely	2 (10.53)
	Very likely	12 (63.16)

Abbreviations: DBP, diastolic blood pressure; SBP, systolic blood pressure.
